# Supplementary material for: Bacillus subtilis Response to Mercury Toxicity: A Defense Mediated by Sulphur-Rich Molecules and Oxidative Prevention Systems
Source: Int J Mol Sci. 2025 Oct 20;26(20):10179. doi: 10.3390/ijms262010179 (PMC12563154; doi:10.3390/ijms262010179)
Supplement: Supplementary file 1 [file ijms-26-10179-s001.zip › ijms-3852128-supplementary.pdf]

Supplementary Material

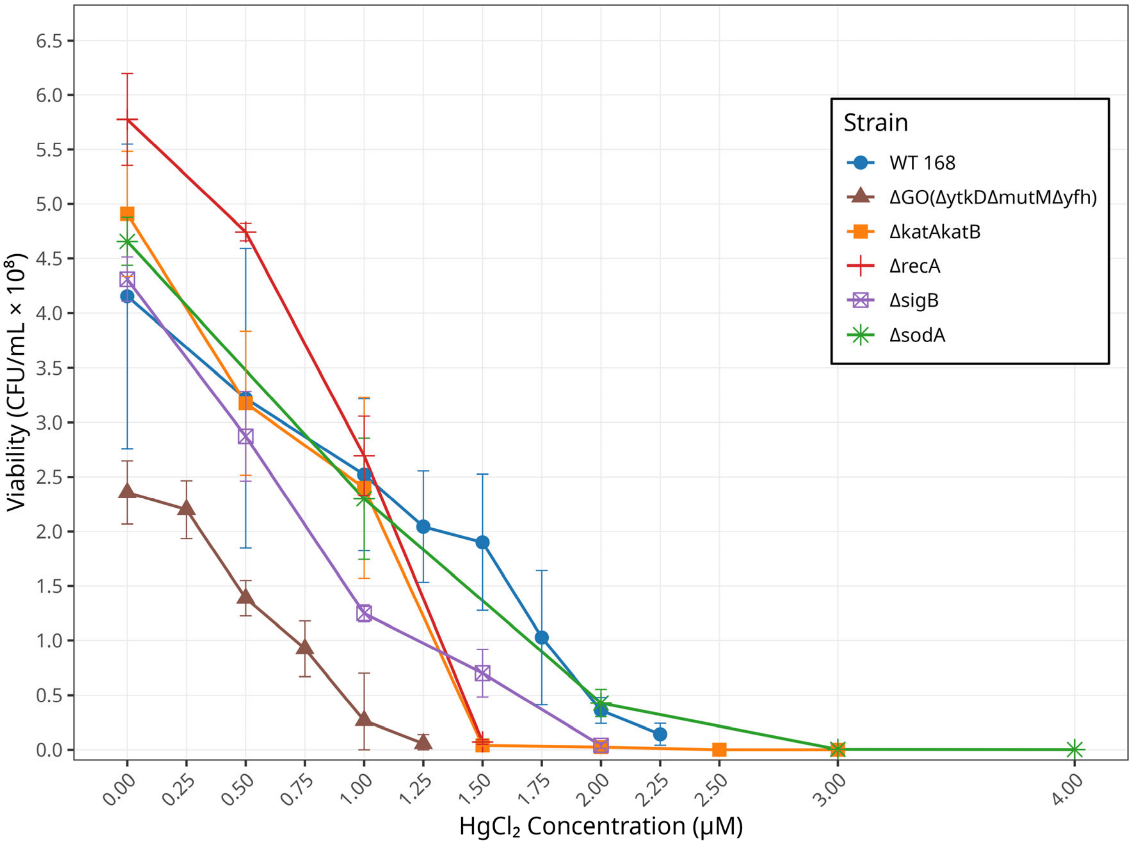

Figure S1. Determination of LD_{50} and LD_{90} of $HgCl_2$ in different *Bacillus subtilis* strains. The effect of $HgCl_2$ over the viability of wild type *B. subtilis* 168 cells and *ΔrecA*, *ΔkatAkatB*, *ΔsigB*, *ΔsodA* and ΔGO mutants was analyzed by determination of the LD_{50} and LD_{90}. Plots show the cell viability as Colony Forming Units per milliliter (CFU/mL) as a function of the $HgCl_2$ concentration. LD_{50} and LD_{90} of $HgCl_2$ were calculated from the equation obtained from linear regression (straight dotted line). The mean ± SD of three independent experiments is shown.

Table S1. Upregulated genes of *Bacillus subtilis* in response to the Hg(II) ions exposure.

	gene	logFC	logCPM	PValue	FDR	Description
1	*tcyC*	1.26283612	8.694601336	0.000422335	0.018282312	cystine ABC transporter (ATP-binding protein)
2	*tcyB*	1.431142474	8.298062205	8.02E-05	0.004615442	cystine ABC transporter (permease)
3	*aseR*	1.876496467	2.128350034	0.000184921	0.009028877	transcriptional regulator (metals sensing ArsR-SmtB repressors family)
4	*arsF*	1.557194095	5.76729009	0.000256125	0.011441172	arsenite/antimonite/H+ antiporter
5	*ydgK*	1.622696019	4.292331084	0.000188291	0.009087755	putative efflux transporter
6	*ydhK*	2.591631768	8.189687122	1.62E-05	0.001304651	hypothetical protein BSU_05790
7	*tcyP*	1.258122595	9.520839565	0.000756627	0.02910959	(sodium)-cystine symporter
8	*nrdI*	2.640771028	4.469573287	3.34E-07	4.52E-05	co-factor of ribonucleotide diphosphate reductase
9	*nrdE*	3.007763766	9.936983476	9.80E-16	5.88E-13	ribonucleoside-diphosphate reductase (major subunit)
10	*nrdF*	2.873379772	9.557335215	1.36E-14	7.13E-12	ribonucleoside-diphosphate reductase (minor subunit)
11	*ymaB*	2.310435239	6.579939874	2.53E-08	5.31E-06	putative cofactor involved in deoxyribonucleotide synthesis
12	*yomN*	3.805895112	-0.223456981	7.93E-05	0.004615442	conserved protein of unknown function; phage SPbeta
13	*arsC*	2.240257179	6.619913735	2.89E-05	0.001987538	thioredoxin-coupled arsenate reductase; skin element
14	*arsB*	1.955477839	7.946200789	4.30E-06	0.000400866	arsenite efflux transporter; skin element
15	*yrkH*	2.265002383	3.074105794	0.00017116	0.008555941	putative sulfur transferase / hydrolase
16	*yrkF*	2.40172839	2.176688041	0.000137188	0.007291778	putative rhodanese-related sulfur transferase
17	*yrkE*	3.748484772	1.003414153	0.00135904	0.049194907	putative protein involved in sulfur metabolism (DsrE-like)
18	*ytnM*	1.479907198	5.017315089	0.000762576	0.02910959	putative transporter
19	*tcyN*	1.567449819	6.31263953	0.000415297	0.018164936	sulfur-containing amino-acid ABC transporter (ATP-binding protein)
20	*tcyM*	1.810938578	5.69368206	8.55E-05	0.00478698	sulfur-containing amino acid ABC transporter (permease)
21	*tcyL*	1.643905412	5.843738652	0.000190807	0.009104549	sulfur-containing amino acid ABC transporter (permease)
22	*tcyK*	1.571775884	6.302575818	0.00043827	0.018402965	sulfur-containing amino acid

						ABC transporter binding lipoprotein
23	*tcyJ*	1.828797231	5.350556611	0.000143633	0.007538927	sulfur containing amino acid ABC transporter binding lipoprotein
24	*yugS*	1.734223698	4.535018208	8.21E-05	0.004661425	putative membrane protein
25	*copA*	2.487544064	12.66132019	2.32E-10	8.84E-08	copper transporter ATPase
26	*yvdD*	1.868958767	6.553771715	1.72E-05	0.001365723	putative enzyme
27	*tkmA*	2.040701419	4.967779719	3.55E-06	0.000363976	modulator of PtkA protein tyrosine kinase activity; modulation of biofilm formation
28	*yybF*	1.810263866	4.443822681	4.39E-05	0.002838255	putative permease

Supplementary Table S2. Downregulated genes of *Bacillus subtilis* in response to the Hg(II) ions exposure.

	gene	logFC	logCPM	PValue	FDR	Description
1	*eesA*	-2.197563303	5.096134044	1.81E-06	0.000194682	iron-chelator (enterobactin family) esterase
2	*feuC*	-1.570940186	6.32712656	0.000151672	0.00776674	iron-uptake protein
3	*btr*	-1.781258141	5.729362974	2.41E-05	0.001688109	transcriptional activator (AraC/XylS family) of synthesis and uptake of the siderophore bacillibactin
4	*ybfP*	-1.752532278	6.349647718	5.58E-05	0.003546927	putative transcriptional regulator (AraC/XylS family)
5	*yceC*	-1.745390045	10.26440381	1.40E-06	0.000156063	putative stress adaptation protein (tellurite resistance)
6	*yceD*	-1.59079942	11.33105231	1.84E-05	0.001403698	putative stress adaptation protein (tellurite resistance)
7	*yceE*	-1.358177097	11.36586931	0.000198057	0.009240444	putative stress adaptation protein (tellurite resistance)
8	*yceF*	-1.240540748	11.32724028	0.000512163	0.020678584	putative stress adaptation transporter (tellurite resistance)
9	*yceG*	-1.369364702	10.03072778	0.000222366	0.010039931	putative toxic compound adaptation protein (tellurite resistance)
10	*yceH*	-1.394914117	9.507210294	0.000146066	0.00757197	putative reactive oxygen species resistance protein
11	*ffoR*	-2.114089494	6.272767559	7.93E-07	0.000100909	Fur-regulated NADPH:ferredoxin oxidoreductase
12	*pbtO*	-1.774926923	6.025917271	9.60E-06	0.000822982	petrobactin iron-siderophore ABC transporter (permease)
13	*pbtP*	-2.445390732	6.342454793	8.60E-09	2.01E-06	petrobactin iron-siderophore ABC transporter (ATP-

						binding protein)
14	*pbtQ*	-2.284869411	7.60833273	2.99E-09	7.84E-07	petrobactin iron-siderophore ABC transporter (binding lipoprotein)
15	*ydbS*	-1.283887094	7.873735831	0.000951285	0.035664685	resistance to heterologous antibiotics
16	*ydbT*	-1.972984482	9.73997259	1.12E-06	0.000133944	resistance to heterologous antibiotics
17	*ydcC*	-1.526907356	6.651940628	0.000154669	0.007824771	putative lipoprotein
18	*ydgG*	-4.068151446	3.668841238	2.35E-09	7.04E-07	putative transcriptional regulator (MarR family)
19	*ydgH*	-4.82017814	10.64040866	3.91E-18	2.74E-15	putative membrane component
20	*pspA*	-2.675395095	7.275456841	1.36E-07	2.12E-05	phage shock protein A homolog regulator; prophage region 3
21	*ydjG*	-1.803041528	7.594325555	6.53E-06	0.000570987	putative phage replication protein; prophage region 3
22	*ydjH*	-1.884518623	7.218489912	5.05E-06	0.000450856	conserved hypothetical protein; prophage region 3
23	*ydjI*	-2.092293002	9.482240541	3.56E-08	7.11E-06	putative phage protein
24	*ydjJ*	-2.020488237	2.354508086	3.55E-05	0.002365284	putative membrane associated potassium channel; prophage region 3
25	*ydjP*	-1.491040039	8.862518445	9.51E-05	0.005254271	putative aminoacrylate hydrolase
26	*yeaA*	-1.341284943	8.560477108	0.000195287	0.009213596	conserved hypothetical protein
27	*hmoA*	-2.633941231	6.806398671	1.55E-09	5.42E-07	heme-degrading monooxygenase
28	*sxzA*	-1.414891987	6.51379258	0.000429664	0.018338296	xenosiderophore schizokinen (dihydroxamate) transporter (permease)
29	*yfhC*	-1.698148657	7.092647006	2.37E-05	0.001685909	putative oxidoreductase (nitroreductase family)
30	*spo0M*	-2.044186714	11.0200619	8.29E-08	1.41E-05	protein involved in the control of the cell cycle as a function of the environment
31	*yjoB*	-1.881478703	9.343024407	1.56E-07	2.33E-05	informational ATPase possibly involved in protein degradation
32	*aeeB*	-1.686015873	3.530601617	0.000557164	0.021864777	L-Ala-D/L-Glu epimerase
33	*fldN*	-3.627477824	4.578526258	3.27E-13	1.53E-10	short-chain flavodoxin (acts in lipid desaturation)
34	*ykuO*	-4.392813038	6.15142288	2.89E-19	2.42E-16	conserved hypothetical protein
35	*fldP*	-4.790447223	5.078799239	2.45E-19	2.42E-16	short-chain flavodoxin
36	*skiW*	-1.881047376	7.774499163	1.41E-06	0.000156063	subunit of permease

						exporting the starvation-induced killing protein
37	*skiX*	-1.809029802	7.357182225	7.36E-05	0.004417144	subunit of efflux permease exporting the starvation-induced killing protein
38	*skiY*	-1.687394416	7.399837065	6.97E-05	0.004299612	subunit of efflux permease exporting the starvation-induced killing protein (ATP-binding protein)
39	*skiZ*	-1.587780138	9.397833906	2.33E-05	0.001685909	permease subunit exporting Sporulation-Delaying Protein
40	*fosB*	-1.582474712	6.930996628	0.000180595	0.008921383	magnesium-dependent bacillithiol-transferase
41	*yzzP*	-1.754205406	6.119348559	3.75E-05	0.002460829	conserved protein of unknown function
42	*desE*	-1.634219454	7.641620034	1.37E-05	0.001126864	fatty acid desaturase
43	*yocL*	-2.979237059	-0.029363976	0.000220455	0.010039931	conserved protein of unknown function
44	*yqfB*	-1.52140522	9.354436731	1.91E-05	0.001431207	conserved protein of unknown function
45	*floA*	-1.470157574	10.3774061	3.02E-05	0.002044482	flotillin-like protein involved in membrane lipid rafts
46	*bscR*	-1.492722491	5.051471855	0.000482573	0.019673067	transcriptional regulator for cypB
47	*nifS*	-2.202179291	2.297148154	0.000432363	0.018338296	desulfurase involved in iron-sulfur clusters for NAD biosynthesis
48	*pftB*	-2.213110189	7.306520379	2.11E-08	4.66E-06	pyruvate import system subunit B
49	*pftA*	-2.552466363	5.820271588	5.67E-09	1.40E-06	pyruvate uptake system subunit A
50	*yteJ*	-1.632233007	9.09763844	2.01E-05	0.001483876	putative integral inner membrane protein
51	*sppA*	-1.575372647	9.004478051	6.34E-05	0.003973738	signal peptide peptidase
52	*ythQ*	-1.548585266	8.784540311	7.76E-05	0.004589243	putative ABC transporter (permease)
53	*dhbB*	-2.023806395	8.570693774	1.16E-06	0.00013496	isochorismatase (siderophore specific)
54	*dhbE*	-2.33775236	8.896358999	3.09E-07	4.32E-05	2,3-dihydroxybenzoate-AMP ligase
55	*dhbC*	-2.452483551	8.717769459	8.40E-08	1.41E-05	isochorismate synthase (siderophore-specific)
56	*dhbA*	-2.904182569	7.560562416	1.81E-09	5.85E-07	2,3-dihydro-2,3-dihydroxybenzoate dehydrogenase
57	*besA*	-2.075589021	6.885664131	8.53E-07	0.000105351	bacillibactin trilactone hydrolase

58	*feuV*	-1.285580795	6.031012315	0.001319894	0.048193348	iron(III)-siderophore transporter (ATP binding component)
59	*liaH*	-2.097246003	6.715893373	0.000482407	0.019673067	modulator of liaIHGFSR (yvqIHGFEC) operon expression
60	*liaI*	-2.196442002	5.369861107	0.000531409	0.021251312	membrane anchor for the phage-shock protein A homolog LiaH
61	*fhuC*	-2.795341421	6.217792777	6.69E-11	2.81E-08	ferrichrome ABC transporter (ATP-binding protein)
62	*fhuG*	-2.304843005	5.565193542	1.00E-07	1.62E-05	ferrichrome ABC transporter (permease)
63	*modA*	-2.985849655	6.541290928	1.72E-07	2.49E-05	molybdate-binding lipoprotein
64	*modB*	-2.669012063	6.310908984	4.05E-06	0.00038624	molybdenum transport permease
65	*yvaC*	-2.921094906	4.514298394	2.75E-09	7.69E-07	putative integral inner membrane protein
66	*yvaD*	-4.818046918	7.845339782	2.74E-28	5.75E-25	putative integral inner membrane protein
67	*yvaE*	-4.94495093	7.568061255	8.98E-25	1.26E-21	putative metabolite-efflux transporter
68	*yvaF*	-5.253032533	8.395873336	3.27E-33	1.37E-29	putative transcriptional regulator
69	*padC*	-1.876949978	4.345765822	0.000546675	0.021655558	phenolic acid decarboxylase
70	*racX*	-2.382828341	9.803191452	3.92E-08	7.49E-06	promiscuous aminoacid racemase (prefers arginine, lysine and ornithine)
71	*pbpE*	-2.315788757	10.20492488	4.39E-08	8.02E-06	penicillin-binding protein 4*
72	*yvdT*	-2.569822829	7.83341744	1.82E-05	0.001403698	putative transcriptional regulator (TetR/AcrR family)
73	*psmA*	-4.306434515	5.30435519	1.92E-06	0.000201169	sodium / proton antiporter subunit A
74	*psmB*	-4.315646234	5.574634291	3.60E-07	4.72E-05	sodium-proton two component antiporter subunit
75	*yvdQ*	-2.880188061	2.703327196	0.000115479	0.006216636	conserved protein of unknown function
76	*yvlD*	-1.791989035	8.391004227	4.68E-06	0.000427548	putative integral phage holin-like membrane protein
77	*yvlC*	-1.86814539	6.787084779	1.17E-05	0.000981373	membrane associated phage-like stress regulator, nisin resistance
78	*yvlB*	-1.760282771	10.10239733	3.68E-06	0.000368398	conserved protein of unknown function, stress-related
79	*yvlA*	-1.579651397	5.609192008	0.000364479	0.016109952	conserved protein of

						unknown function
80	*csbA*	-1.464148199	3.675970622	0.001051093	0.038715263	conserved membrane protein of unknown function
81	*efeB*	-1.396209432	8.127253595	0.000212575	0.009808809	peroxidase converting ferric iron into ferrous iron
82	*efeM*	-1.533140488	7.476710755	0.000112964	0.006160219	lipoprotein binding ferrous or ferric iron for transport
83	*yxlE*	-1.795528785	3.454570915	0.000942239	0.035643813	negative regulator of sigma-Y activity
84	*yxzE*	-1.566414764	4.255803117	0.000482347	0.019673067	putative bacteriocin
85	*msmX*	-1.688890892	8.313265463	0.000754657	0.02910959	multiple sugar (maltodextrins) transporter ATP-binding protein
86	*yxjI*	-1.932460113	7.481401689	3.92E-06	0.000382896	conserved protein of unknown function
87	*frxB*	-1.759125169	7.673013295	7.07E-05	0.004299612	desferrioxamine-and ferrichrome-binding transporter lipoprotein (shuttle system)
88	*ahpC*	-1.178406799	10.10669704	0.0010455	0.038715263	alkyl hydroperoxide reductase (small subunit)
